# Prognostic nomogram and risk factors for predicting survival in patients with pT2N0M0 esophageal squamous carcinoma

**DOI:** 10.1038/s41598-023-32171-w

**Published:** 2023-03-26

**Authors:** Mei Kang, Yichun Wang, Mingwei Yang, Xiumei Wang, Liyang Zhu, Mei Zhang

**Affiliations:** 1 Department of Radiation Oncology, The First Affiliated Hospital of Anhui Medical University, No.218, Jixi Road, Hefei, 230022 Anhui People’s Republic of China; 2 Department of Oncology, The Third People’s Hospital of Hefei, No. 204, Wangjiang East Road, Baohe District, Hefei, 230022 Anhui People’s Republic of China; 3 Department of Integrated Traditional and Western Medicine Oncology, The First Affiliated Hospital of Anhui Medical University, No.218, Jixi Road, Hefei, 230022 Anhui People’s Republic of China

**Keywords:** Oesophageal cancer, Cancer models

## Abstract

This study analyzed the impact of factors affecting overall survival in patients with pT2N0M0 esophageal squamous carcinoma (ESCC) and developed a nomogram to predict overall survival (OS). We reviewed the clinical data of 413 patients with pathological T2N0M0 ESCC after radical esophagectomy in two hospitals. Data from one institution was used as the training cohort. A nomogram was established using Cox proportional hazard regression for identifying the prognostic factors affecting for OS in ESCC patients. The area under the curve (AUC), calibration curves and decision curve analysis (DCA) were used to evaluate prognostic efficacy, which was validated in an independent validation cohort. In the training cohort (N = 304), the median OS was 69.33 months, and the 3-, 5- and 10-year OS rates were 76.80%, 67.00% and 56.90%, respectively. The median OS of the validation cohort (N = 109) was 73.50 months, and the 3-, 5- and 10-year OS rates were 77.00%, 67.80% and 55.60%, respectively. According to Cox univariate and multivariate analyses, sex, age, tumor length and the number of resected lymph nodes were identified as predictors of OS. We developed nomograms and performed internal and external validation. The time-dependent receiver operating characteristic (ROC) curve and area under the curve (AUC) value, calibration curve and decision curve analysis (DCA) showed good prediction ability of the nomogram. The developed nomogram can effectively predict OS after esophagectomy in patients with pT2N0M0 ESCC.

## Introduction

Esophageal carcinoma (EC) is an aggressive malignancy with poor prognosis and is the sixth leading cause of cancer death and the eighth most diagnosed cancer worldwide, and its incidence has been increasing over the past several decades^1,2^. Esophageal squamous cell carcinoma (ESCC) is the most common pathological subtype of esophageal cancer in China, accounting for more than 90% of all esophageal cancer cases^3^. With the improvement of diagnostic accuracy and medical technology, the prognosis of EC has been significantly improved, but is still unsatisfactory. Surgery is the most important treatment of choice in localized early EC (T1b‐T2 N0‐1 and M0), the failure of postoperative treatment is mainly due to regional recurrence and distant metastasis^4^. At present, there are few progresses in the research on the stage, treatment and prognosis of stage T2 ESCC. Numerous studies indicated that tumor grade, tumor infiltration depth and lymph node metastasis are important prognostic indicators for T2 stage EC^5^. The optimum treatment strategy for T2 stage EC has not been determined. The systematic comprehensive treatment mode of preoperative neoadjuvant therapy combined with surgery have attracted more and more attention of clinicians^6,7^. A retrospective study with T2 stage EC patients, the 5-year survival rate of patients was 64.1%, which was improved compared with surgery^8^. However, there is still no evidence to prove that there is a significant difference between preoperative treatment and surgery in resection rate, recurrence rate and long-term survival rate.

This study aimed to analyze the relationship between prognostic factors and overall survival (OS) in resected cases of pT2N0M0 ESCC from the First Affiliated Hospital of Anhui Medical University and Hefei Third People’s Hospital through a database to establish a prognostic nomogram for ESCC. Based on the nomogram, related factors affecting the prognosis of EC patients were screened to predict the survival rate. This may be valuable to clinicians for improved treatment decisions to improve clinical outcomes.

## Patients selection and methods

### Patient selection

The complete clinicopathological data of 304 previously untreated EC patients who underwent radical esophagectomy were obtained from the First Affiliated Hospital of Anhui Medical University and 109 patients from the Hefei Third People's Hospital between January 2010 and March 2019 were retrospectively analyzed.

The inclusion and exclusion criteria for enrolled patients were as follows: (I) pathological diagnosis was squamous cell carcinoma; (II) underwent radical surgery; (III) pT2N0M0 (staging according to TNM 8th edition); (IV) whose complete postoperative information was available. The exclusion criteria were as follows: (I) age < 18 years; (II) lack of complete clinicopathological or follow-up data; and (III) previous history of other tumors or secondary primary tumors.

Cases from the First Affiliated Hospital of Anhui Medical University served as the training cohort, and those from the Hefei Third People's Hospital served as the validation cohort.

Written informed consent was waived due to the retrospective design and the absence of any intervention.

### Data collection

We retrospectively collected the demographic characteristics and clinicopathological characteristics of 413 patients from the two hospitals, including age, sex, tumor location, tumor length, tumor grade, the number of lymph nodes dissected, chemotherapy and radiotherapy. The patients were followed up every 3 months during the first 2 years after the operation, every 6 months after 2 years, and every year after 5 years. Follow-up examinations included routine laboratory examinations, computed tomography (CT), ultrasound of superficial lymph nodes, barium meal of the upper digestive tract and/or positron emission tomography (PET)/CT.

The primary study outcome was OS, which was calculated from the date of esophagectomy to the date of death or the last follow-up. Patients with a survival time of 0 months were excluded. Survival status was determined by querying patient hospitalization data and telephone follow-up. The end time of follow-up is the October 2021.

According to the Ministry of Health  (Ethics review on biomedical research involving human subjects), WMA  (Declarations of Helsinki) and CIOMS  (International ethical guidelines for biomedical research involving), all methods were performed in accordance with the relevant guidelines and regulations.

### Statistical analysis

Statistical analysis was conducted using IBM SPSS Statistics (version 20.0, USA) and R language version 4.1.1. All clinicopathological factors were transformed into categorical variables based on the cutoff values determined by the X-tile software 3.6.1. All time events were estimated using the Kaplan–Meier method and compared using log-rank tests. We used univariate and multivariate Cox proportional hazard regression-adjusted potential confounding variables to calculate adjusted risk ratios (HRs) and associated 95% confidence intervals (CIs). The proportional hazard assumption was assessed using Cox models that allowed time-dependent HRs combined with a curve of S (t) * log [- log (t)]. P-values < 0.05 were considered statistically significant.

Multivariate analyses were applied to identify prognostic factors, and the nomogram was developed with corresponding values given the selected prognostic factors. Each independent prognostic factor in the nomogram was assigned a score, and the total score was calculated from the patient data to predict the 3-, 5-, and 10-year OS rates.

Next, we used the bootstrap method to use internal validation to estimate the prediction accuracy of the nomogram, which was presented as the time-dependent receiver operating characteristic (ROC) curve and the area under the curve (AUC) value. The calibration curve and decision curve analysis (DCA) were used to verify the prediction effect of the model. Then, we performed validation training for external validation to evaluate the performance of the prediction model.

### Ethics approval and consent to participate

This study was approved by the Ethics Committee of the First Affiliated Hospital of Anhui Medical University and the Third People's Hospital of Hefei City and agreed to be published.

## Results

### Descriptive statistics

The baseline characteristics are summarized in Table 1. Between January 2010 and March 2019, a total of 413 pT2N0M0 ESCC patients were recruited from the First Affiliated Hospital of Anhui Medical University (training cohort, n = 304) and the Hefei Third People's Hospital (validation cohort, n = 109). The patients included more males than females (75.79% vs. 24.21%), and the median age was 65 years (range, 37–78 years). More than half of the patients had primary tumors located in the middle of the esophagus (66.10%), with a median tumor length of 3 cm, and tumor grade mostly well differentiated (moderate to well differentiation, 73.37%). Less than 10 lymph nodes were removed in 63.20% of the patients. A total of 14.29% of patients received adjuvant radiotherapy, and 20.58% received adjuvant chemotherapy. The demographic and clinical factors were largely consistent between the training and validation cohorts.

**Table 1:** Baseline characteristics of pT2N0M0 patients with ESCC.

Characteristics	Total (n = 413)	Training cohort (n = 304)	Validation cohort (n = 109)
	No. of patients (%)	No. of patients (%)	No. of patients (%)
Age			
< 65	208 (50.36)	155 (50.99)	53 (48.62)
≥ 65	205 (49.64)	149 (49.01)	56 (51.38)
Gender			
Female	100 (24.21)	68 (22.37)	32 (29.36)
Male	313 (75.79)	236 (77.63)	77 (70.64)
Location			
Upper	27 (6.54)	23 (7.57)	4 (3.67)
Middle	273 (66.10)	190 (62.50)	83 (76.15)
Low	113 (27.36)	91 (29.93)	22 (20.18)
Grade			
Poor	110 (26.63)	85 (27.96)	25 (22.94)
Well	303 (73.37)	219 (72.04)	84 (77.06)
Length (cm)			
> 2	342 (82.81)	249 (81.91)	93 (85.32)
≤ 2	71 (17.19)	55 (18.09)	16 (14.86)
Lymph node harvested			
> 10	152 (36.80)	112 (36.84)	40 (36.70)
≤ 10	261 (63.20)	192 (63.16)	69 (63.30)
Radiotherapy			
No	354 (85.71)	260 (85.53)	94 (84.24)
Yes	59 (14.29)	44 (14.47)	15 (13.76)
Chemotherapy			
No	308 (74.58)	229 (75.32)	79 (72.48)
Yes	85 (20.58)	75 (24.67)	30 (27.52)

The median OS of the training cohort was 69.33 months, and the 3-, 5-, and 10-year OS rates were 76.80%, 67.00%, and 56.90%, respectively. The median OS in the validation cohort was 73.50 months, and the 3-, 5-, and 10-year OS rates were 77.00%, 67.80%, and 55.60%, respectively.

### Cox regression analysis

**Figure 1:**
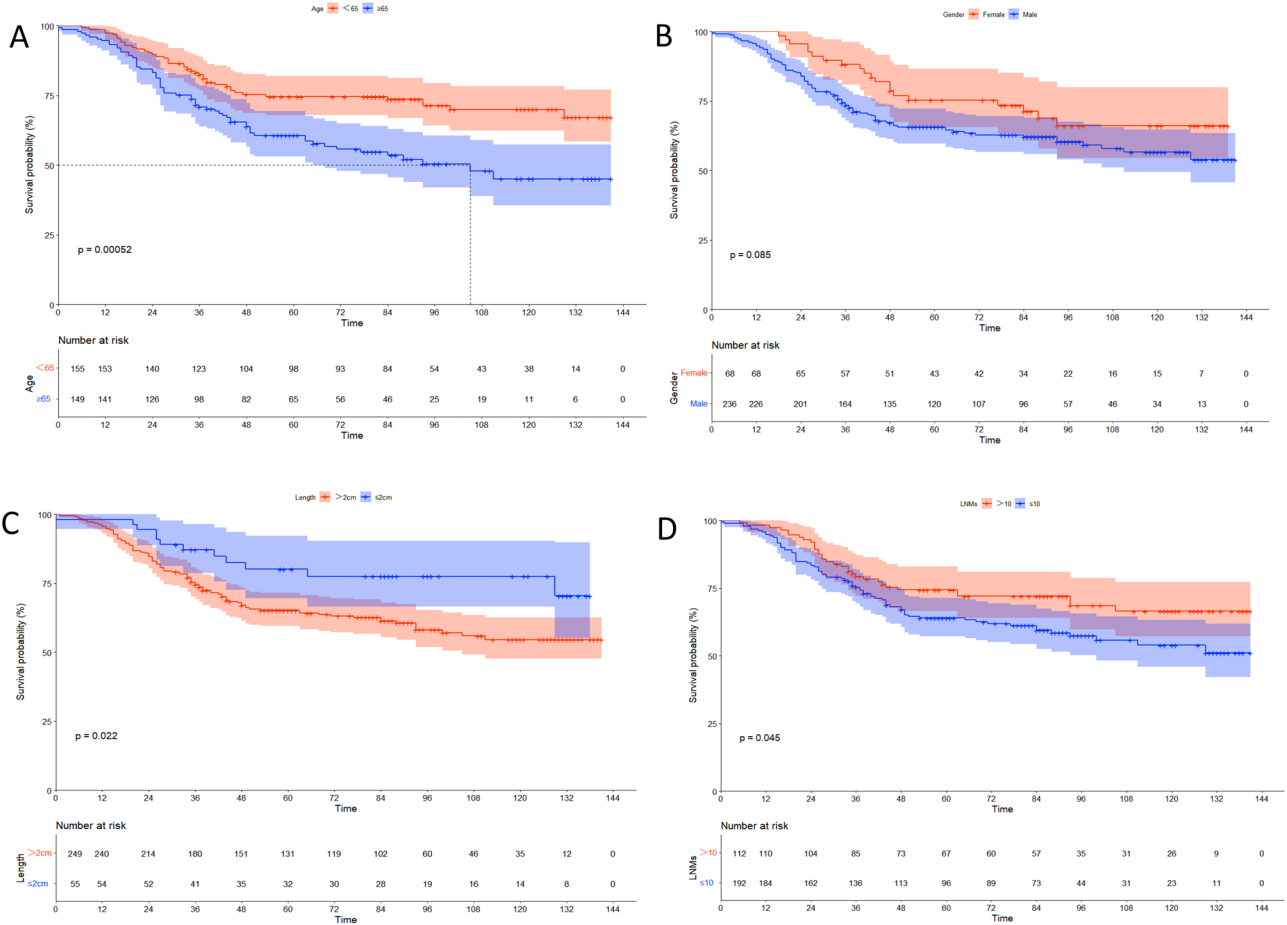
Kaplan–Meier curves for the OS of the training cohort. Age group (**A**), sex group (**B**), length group (**C**), lymph node metastasis (LNM) group (**D**).

In the univariate Cox analysis, sex, tumor length, and the number of lymph nodes harvested were prognostic predictors of OS, and the primary location, differentiation, postoperative adjuvant radiotherapy or chemotherapy did not affect the prognosis. Multivariate analysis showed that the male sex, age over 65 years, tumor length > 2 cm, and the number of harvested lymph nodes < 10 were independent factors affecting OS (Table 2). Finally, a survival curve of those risk factors was displayed using the Kaplan–Meier method suing R software (Fig. 1).

**Table 2:** Univariate and multivariate Cox proportional hazard model analysis in the training cohort.

Characteristic	Univariate analysis			Multivariate analysis		
	HR	95% CI	P value	HR	95% CI	P value
Age			P < 0.001			0.002
< 65	Ref			Ref		
≥ 65	1.955	1.330–2.874		1.877	1.271–2.772	
Gender			0.085			0.021
Female	Ref			Ref		
Male	1.530	0.939–2.471		1.781	1.091–2.908	
Location			0.630			
Upper	Ref					
Middle	0.803	0.401–1.612				
Low	0.971	0.465–2.025				
Grade			0.188			
Poor	Ref					
Well	0.765	0.511–1.141				
Length (cm)			0.022			0.031
> 2	Ref			Ref		
≤ 2	0.502	0.276–0.915		0.515	0.282–0.941	
Lymph node harvested			0.045			0.042
> 10	Ref			Ref		
≤ 10	1.514	1.006–2.277		1.538	1.016–2.328	
Radiotherapy			0.613			
No	Ref					
Yes	1.139	0.687–1.889				
Chemotherapy			0.162			
No	Ref					
Yes	1.338	0.690–2.012

### Nomogram development and internal validation

**Figure 2:**
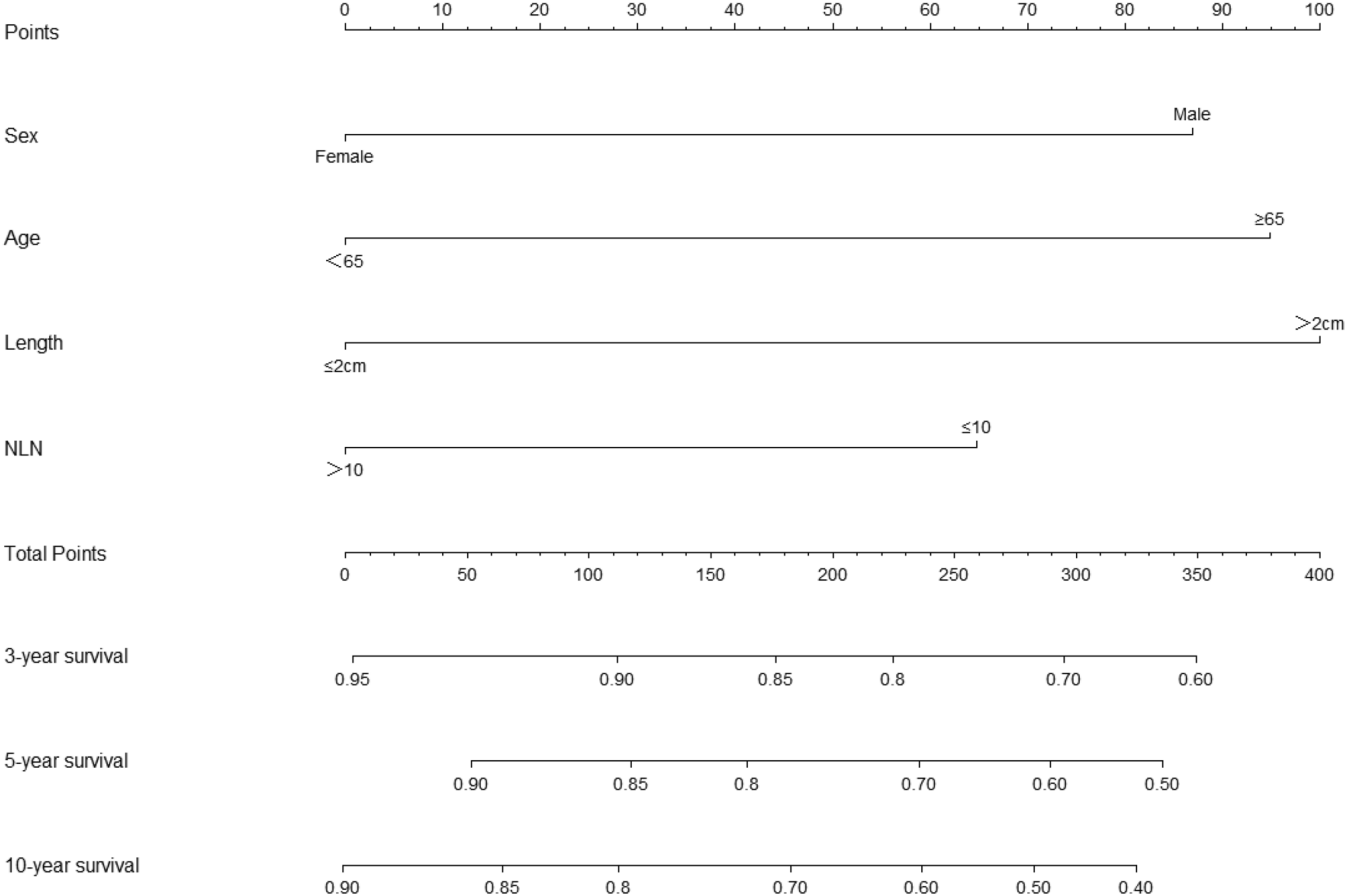
Nomogram predicting the 3-,5- and 10-year OS rates from the training cohort.

Based on the results of multivariate analysis, all independent predictors of OS were integrated to construct a prognostic nomogram, which was used to calculate the OS rates at 3, 5, and 10 years (Fig. 2).

**Figure 3:**
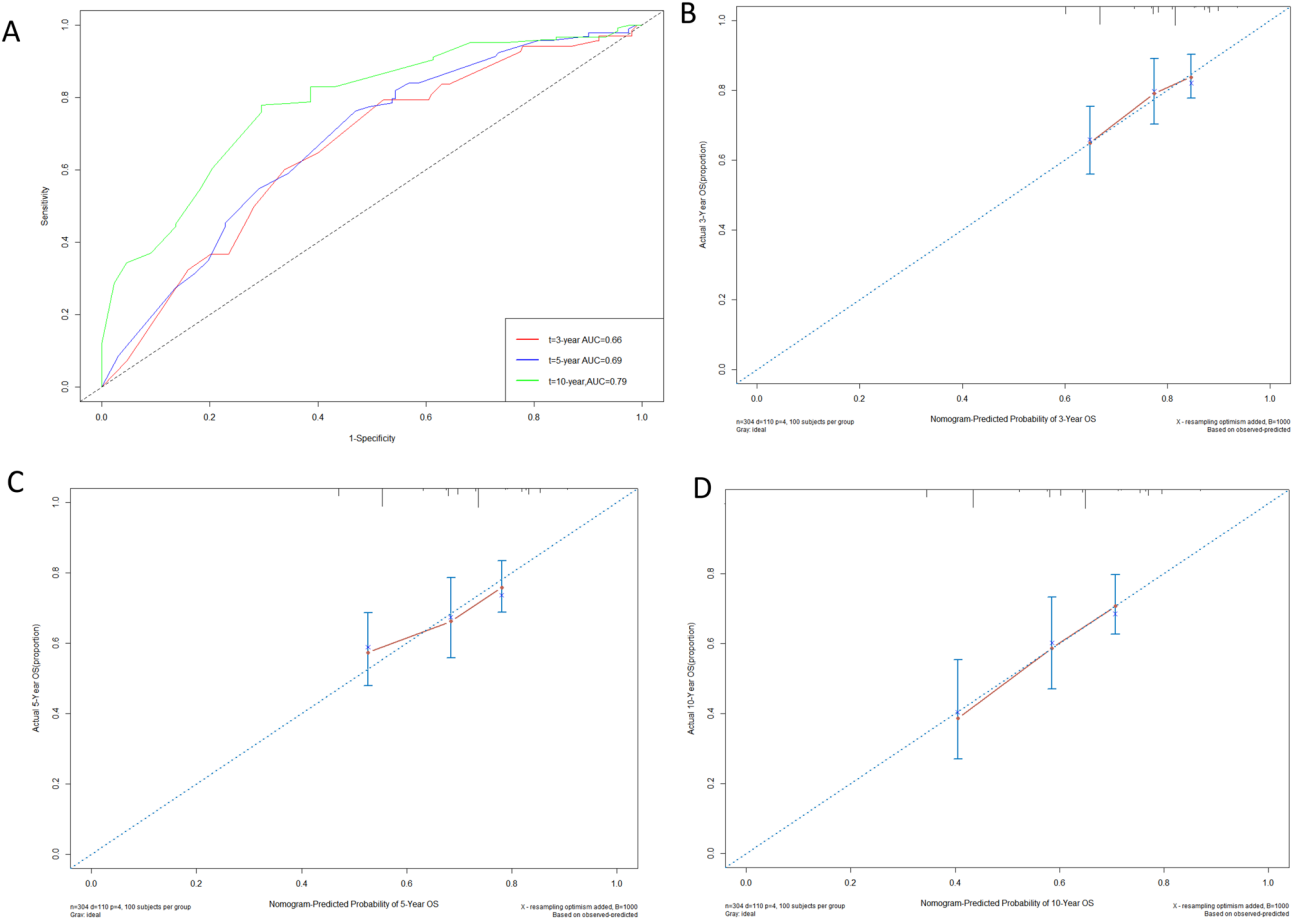
** (A)** ROC curves for 3-, 5-, and 10-year OS based on the nomogram for the training cohort. (**B)** Calibration curve of predicted 3-year OS. (**C)** Calibration curve of the predicted 5-year OS. (**D)** Calibration curve of the predicted 10-year OS.

Then, we performed internal validation of the nomogram and found that the nomogram had a concordance index (C-index) of 0.68. The 3-, 5-, and 10-year AUC values for ROC were 0.66 (95% CI 0.59–0.73), 0.69 (95% CI 0.61–0.74) and 0.79 (95% CI 0.71–0.86), respectively (Fig. 3A). The calibration curves were also applied to verify the predicted effect of the nomogram, and indicated that the calibration plot was highly consistent between the predicted survival and actual survival rates (Fig. 3B–D).

### External validation

**Figure 4:**
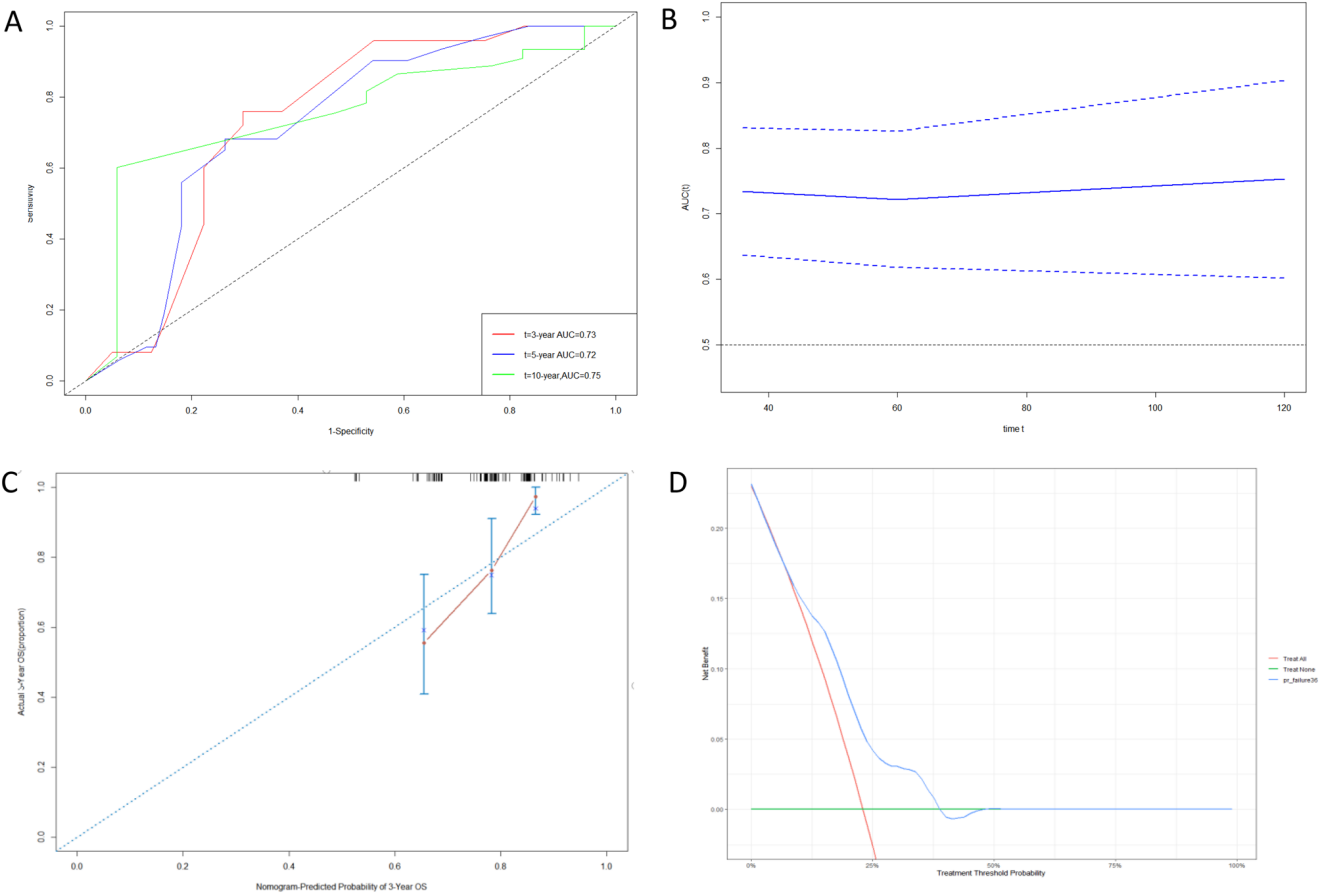
** (A)** ROC curves for 3-, 5-, and 10-year OS based on the nomogram for the validation cohort. (**B)** Time-dependent AUC curve showing the performance of the nomogram in predicting OS in the validation cohort. (**C)** Calibration curve of the predicted 3-year OS. (**D)** Decision curve analysis for 3-year OS.

The nomogram was externally validated using an independent validation cohort. The C-index of the nomogram was 0.69. The 3-, 5-, and 10-year AUC values for ROC were 0.73 (95% CI 0.64–0.83), 0.72 (95% CI 0.62–0.83) and 0.75 (95% CI 0.61–0.90), respectively (Fig. 4A), and the time-dependent AUC curve showing the performance of the nomogram in predicting OS in the validation cohort was plotted (Fig. 4B). Considering the 3-year OS rate, the calibration plot showed good conformity between the predicted and actual probability for OS (Fig. 4C). Finally, we performed DCA to assess the clinical utility of the nomogram, suggesting that was clinically valuable in predicting survival (Fig. 4D).

## Discussion

In recent decades, the prognosis of EC has gradually improved in many countries. EC treatment ranges from simple surgery to surgery-based comprehensive treatment and from postoperative adjuvant therapy to preoperative neoadjuvant therapy; however, the treatment of T2 stage ESCC is still inconclusive. Compared with other stages, the treatment and prognosis of T2 ESCC are more controversial. Previous studies have shown that many clinicopathological factors are associated with the prognosis of ESCC^9,10^. As we retrospectively analyzed 413 patients with pathological T2N0 from two different hospitals. The results showed that some factors, namely the male sex, age over 60 years, tumor length > 2 cm, and the number of intraoperative lymph nodes dissected < 10 affected survival while, the degree of differentiation, tumor location and postoperative adjuvant therapy had no significance on prognosis (Table 2). In the present study, sex was an independent risk factor affecting OS after T2N0 ESCC in that the survival rate of females was significantly higher than that of males. Previous studies have also reported that the survival rate of women after esophageal cancer is higher, and may be related to the poor lifestyle habits, such as smoking and drinking, of male patients^11,12^. Similarly, We found that age also affects survival after esophageal cancer surgery. The poor nutritional status, decreased body resistance, and difficulty in recovering from surgery associated with older age may affect the prognosis.

Clinicians believe that the differentiation degree of tumor cells can reflect their ability of malignant invasion; the lower the degree of differentiation is, the higher the degree of malignancy, and the likelihood of early metastasis and postoperative recurrence. Several studies have shown that well-differentiated tumors have a better prognosis, while some other studies suggest no significant correlation between tumor differentiation and overall survival^13,14^. Therefore, the association between tumor differentiation and prognosis remains controversial, we found no such correlation in our study. There also exists no consensus on whether tumor location affects postoperative survival in EC^15^; in our study, we found no significance between tumor location and postoperative survival. We also found that the number of lymph nodes harvested during surgery was an independent risk factor for OS, and that prognosis was poor when the number of lymph nodes dissected was < 10. Studies have reported that the more lymph nodes removed during surgery, the better the prognosis, which is in line with our findings^16^. A considerable number of patients with pathological T2N0 stage after radical esophageal cancer have insufficient lymph node dissection, which may affect the prognosis with underestimation of the stage. Therefore, the National Comprehensive Cancer Network (NCCN) guidelines recommend the dissection of at least 15 lymph nodes to allow adequate lymph node staging in patients undergoing esophagectomy.

The retrospective results showed that adjuvant radiotherapy could improve the median survival of patients by 4–6 months, and the 3-year OS rate ranged from 2.9 to 3.3%^17,18^. In the current consensus, adjuvant therapy is not recommended for pathological T2N0 esophageal cancer. In the present study, some patients still received postoperative adjuvant therapy for the following reasons: (1) patient-related factors: the choice of postoperative adjuvant therapy is affected by factors such as patient intention, economy, and physical fitness; (2) surgery-related factors: there is a high possibility of insufficient mediastinal lymph node dissection in surgical operations and routine cervical lymph node dissection is not performed; 63.20% of patients had < 10 lymph nodes dissected in this study, which may affect the accuracy of postoperative staging; (3) oncologist-related factors: due to the poor overall prognosis of esophageal cancer, doctors have high prognostic expectations for patients with early staging and choose adjuvant therapy under the condition of controllable side effects, hoping to improve the survival rate. The study showed that postoperative radiotherapy or chemotherapy for pT2N0 ESCC could not improve survival, which was consistent with previous reports. Clinicians expect a more reasonable and effective treatment plan to further improve the prognosis because of the poor survival rate after surgery alone^19^. Therefore, despite a lack of consensus on postoperative adjuvant therapy, there are still exploratory studies on the choice of adjuvant therapy. Previous randomized controlled trials have shown that postoperative radiotherapy can improve the disease-free survival rate and local–regional recurrence rate of pT2-3N0 patients after radical surgery^20^. Although the OS rate improved, the difference was not statistically significant. Another study also showed that postoperative chemotherapy significantly improves 5-year OS in patients with stage I–III ESCC^21^.

Nomograms serve as reliable tools to assist clinical decision-making and can also be used as a reference for treatment strategies. Nomograms turn complex regression equations into visual graphs with readable results for easy evaluation, and have gradually gained increasing attention and application in medical research because of their intuitive and easy-to-understand characteristics. Nomograms have been reported to predict survival outcomes after radical esophagectomy in patients with EC^22,23^. In the present study, we collected the clinical data of the patients for Cox univariate and multivariate analyses and developed a nomogram based on the four factors of the multivariate analysis. Using the developed nomogram, the OS rate can be predicted based on the clinicopathological characteristics of a specific patient. Nomograms show 3-, 5-, and 10-year OS rates against the total score. Clinical prediction models require internal and external validation to ensure the performance of individual risk predictions^24,25^. Therefore, we divided the data from the two hospitals into training and validation cohorts. The internal validation of the nomogram model and the external validation of the cohorts showed good reliability and accuracy.

The nomogram had some deficiencies. First, it was established based on a retrospective database and was not verified in a prospective data center. There were certain contamination factors and unavoidable treatment bias. For example, the combination of high-risk factors may result in more lymph node resection or systematic adjuvant treatment. In our study, we used an independent validation cohort from another hospital to verify the nomogram, demonstrating its clinical utility. Second, the prognostic factors we included are limited to the common features related to the postoperative pathology of EC and postoperative adjuvant treatment options. The indicators are relatively simple, and some potential features or biomarkers can be considered to further improve the verification standard.

## Conclusion

We developed and validated an individualized survival prediction nomogram for predicting OS in patients with ESCC. We demonstrated that the constructed nomogram showed accuracy and validity in predicting the prognosis of ESCC after esophagectomy, may be used to help identify high-risk patient populations. The nomogram may be valuable in clinical settings and can be further improved.

## Data Availability

The datasets used and/or analyzed during the current study are available from the corresponding author on reasonable request.

## Abbreviations

EC: Esophageal carcinoma
ESCC: Esophageal squamous cell carcinoma
LNM: Lymph node metastasis
OS: Overall survival
CT: Computed tomography
PET: Positron emission tomography
HRs: Hazard ratios
CIs: Confidence intervals
ROC: Receiver operating characteristic
AUC: Area under curve
DCA: Decision curve analysis
NLN: Number of lymph node
